# Computerized Full-Color Assessment for Distinguishing Color Vision Deficiency

**DOI:** 10.3390/diagnostics15222837

**Published:** 2025-11-09

**Authors:** Jin-Cherng Hsu, Chia-Ying Tsai, Chih-Hsuan Shih, Shao-Rong Huang, Hsing-Yu Wu, Yung-Shin Sun

**Affiliations:** 1 Department of Physics, Fu Jen Catholic University, New Taipei City 242062, Taiwan; 054326@mail.fju.edu.tw; 2 Center for Astronautical Physics and Engineering, National Central University, Taoyuan 237209, Taiwan; 3 Department of Medicine, College of Medicine, Fu Jen Catholic University, New Taipei City 242062, Taiwan; 4 Department of Ophthalmology, Fu Jen Catholic University Hospital, New Taipei City 242062, Taiwan; 5 Graduate Institute of Biomedical and Pharmaceutical Science, Fu Jen Catholic University, New Taipei City 242062, Taiwan; 6 Taiwan Space Agency, Hsinchu City 300091, Taiwan; 7 Diopter Precision Taiwan Co., Ltd., Pingzhen Dist., Taoyan 324032, Taiwan; 8 Institute of Space Systems Engineering, National Yang Ming Chiao Tung University, Hsinchu City 300093, Taiwan

**Keywords:** color vision deficiency (CVD), computerized full-color assessment (CFCA), Farnsworth D-15 test, full-color light generation system

## Abstract

**Background/Objectives**: Current methods for diagnosing color vision deficiency (CVD) generally fall into two categories: computer-based tests that lack full-color lighting and non-computer-based tests that provide full-color lighting. Most of these approaches face several limitations, including inaccurate illumination of test samples, inconsistent test durations, learning effects, and the need for highly skilled operators. **Methods**: To address these limitations, this study introduces the Computerized Full-Color Assessment (CFCA) method, which employs a full-color light generation system based on 16 color spectra selected from the classical Farnsworth D-15 (D-15) test. In the CFCA method, each pair of colors generated by the system was presented under software control, and participants indicated within three seconds whether the colors were different. The total test duration was limited to 5 min. The method was validated using 10 normal trichromats and 11 patients with CVDs. **Results**: Results obtained from the CFCA were compared with those from the classical D-15 test using quantitative parameters, including confusion angle (CA) and confusion index (CI). Correlations between the two methods were analyzed. The *p*-values for CA and CI are 0.688 and 0.587, respectively, and the correlation coefficients are 0.821 for CA and 0.884 for CI, indicating a strong and statistically significant correlation. **Conclusions**: The CFCA method provides an accurate, convenient, and efficient tool for diagnosing CVD, with particular advantages for use in young children. It enables an expanded range of color choices beyond the 16 discs of the D-15 test and allows for the generation of individualized visual spectra, which can be applied in the design of customized color-vision-correcting glasses.

## 1. Introduction

Normal color vision is trichromatic, relying on cone cells to distinguish between the three primary colors (red, green, and blue) [1,2,3]. Specifically, S, M, and L cone cells are retinal photoreceptors with peak absorption at approximately 426 nm (short-wavelength), 530 nm (medium-wavelength), and 557 nm (long-wavelength), respectively [2,3]. Color vision deficiency (CVD) may be acquired or congenital [4]. Acquired CVD is often caused by ocular or neurological disease, systemic illness, drugs, or toxins, and unlike congenital forms, it may be asymmetric, progressive, and sometimes reversible [2,5]. Congenital CVD is an inherited condition caused by anomalies in cone photoreceptors, most commonly arising from mutations in genes on the X chromosome, and is characterized by impaired color discrimination that is present from birth and remains stable throughout life [6]. CVD is typically categorized into red-green and blue-yellow deficiencies, with the former, including protanopia and deuteranopia, being more common.

Standard CVD examination methods include the Nagel anomaloscope test, Ishihara test [7], Farnsworth-Munsell 100 Hue (FM-100) test [8], and Farnsworth D-15 test [9]. Despite their widespread use, these tests present several limitations. Except for the Nagel anomaloscope test, the standard protocols require a 6500 K light source with high color rendering; however, examinations are typically conducted under general light sources, leading to potential inaccuracies. The Nagel anomaloscope, designed in 1907, remains the gold standard, but it is technically demanding as it requires knob adjustments and careful interpretation [10]. The Ishihara test, established in 1917, is commonly used in health screenings. In this test, participants are asked to recognize numbers or patterns embedded in circles of colored dots; however, the test is unsuitable for young children who may not yet recognize numbers [11]. In addition, some participants may memorize the patterns, leading to false negatives. The FM-100 and D-15 tests, developed in the 1940s, require professional supervision for administration and evaluation, limiting their practicality in clinical settings. Their complexity also poses challenges for inexperienced evaluators and younger participants. Computer-based color vision testing methods, such as the Waggoner Computerized Color Vision Test (CCVT) [12,13], the ColorDx CCT HD (Konan Medical) [14], and the CAD (Color Assessment and Diagnosis) test [15], use calibrated displays to present color stimuli and assess an individual’s color discrimination ability. While these methods offer advantages like automated scoring, detailed analysis, and standardized testing, they also have notable drawbacks. Their accuracy depends heavily on proper monitor calibration, display quality, and ambient lighting conditions, which can vary across devices and testing environments. In addition, high equipment costs and limited accessibility, compared to traditional printed tests, restrict their widespread clinical and occupational use.

Commercially available colorblind glasses are spectrally selective eyewear designed to enhance color discrimination in individuals with CVDs. They work by using optical filters, such as notch or interference coatings, to reduce spectral overlap between cones, thereby increasing contrast between confusing colors [16,17]. Popular brands include EnChroma^®^, Pilestone^®^, and Vino Optics^®^. While these glasses may improve color differentiation in some cases, they do not restore normal trichromatic vision, and their effectiveness varies by individual and deficiency type [18,19].

To develop more precise, convenient, and user-friendly examination methods, we propose an approach that reproduces 16 colored light sets across the full-color gamut, based on the D-15 test. The proposed Computerized Full-Color Assessment (CFCA) method incorporates a full-color light generation system to produce the desired colors. This method was validated in 10 normal trichromats and 11 individuals with CVDs, with all participants completing the examination within 5 min using the CFCA system. Its performance was evaluated against the classical D-15 test using quantitative measures, including the confusion angle (CA) and confusion index (CI). Statistical analyses were performed to assess these correlations. This study aims to develop and validate the CFCA method to accurately diagnose and classify CVDs. The objective is to overcome the limitations of conventional and computer-based tests by providing precise, non-metameric color illumination, standardized testing duration, and automated analysis. The present method can support a broader range of color stimuli beyond the 16 discs of the D-15 test and, importantly, enables the creation of individualized visual spectra for designing customized colorblind glasses.

## 2. Materials and Methods

### 2.1. Patient Selection

This study adhered to the principles of the Declaration of Helsinki and was approved by the Institutional Review Board of Fu-Jen Catholic University Hospital (No. FJUH110156). Based on the results from the Ishihara test, a total of 10 female normal trichromats and 11 male patients with CVDs were recruited for the experiments. Individuals with eye infections, discomfort, blurry vision, or a history of ophthalmic surgery within the past six months were excluded. Informed consent was obtained from all participants prior to enrollment, and each participant received a copy of the signed consent form for their records. Although data on ocular pathology screening were not available, all participants, including CVD patients and control volunteers, underwent a complete ocular examination and medical history review by an ophthalmologist (Dr. C. -Y. Tsia, Fu-Jen Catholic University Hospital) before testing. All participants demonstrated best-corrected visual acuity of 20/20, normal intraocular pressure, clear cornea, clear lens, clear media, normal optic disc appearance, and an attached retina without macular lesions. Participants also reported no history of systemic diseases such as hypertension (HTN), diabetes mellitus (DM), or other congenital systemic disorders. Any ocular pathology or subnormal visual acuity resulted in exclusion from the study. The demographic and health status information of all participants is presented in Table 1.

### 2.2. Classical D-15 Method

We used the classical D-15 test as a reference for comparison with our customized system, owing to its ability to discriminate across the full-color gamut. The D-15 test comprises 16 colored discs mounted in circular casings, each approximately 13 mm in diameter. The pilot disc (P) serves as the reference color. Participants are instructed to sequentially arrange the remaining discs in hue order by selecting, at each step, the disc that appears perceptually least different from the most recently placed disc. During the D-15 test, a 25 W LED light source with a color temperature of approximately 6500 K and a color rendering index greater than 90% illuminates the discs. The observer is positioned approximately 25 cm from the discs, defined as the “clear vision distance.” From the observer’s viewpoint, the color discs have an average illuminance of 249 lux.

### 2.3. Full-Color Light Generation System

**Figure 1:**
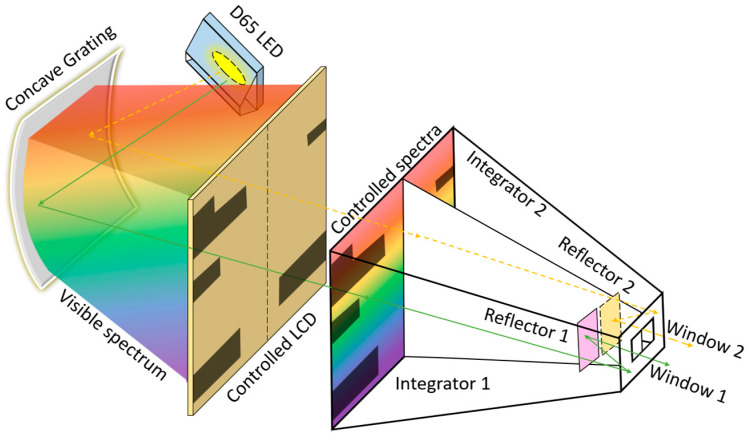
Schematic of the full-color light generation system, which consists of two collimated 25 W D65 LED light sources emitting through a slit and passing through a concave grating to generate two visible spectra when illuminating the two LCD-controlled areas. The spectra are uniformly mixed by Integrators 1 and 2. The mixed lights, reflected by Reflectors 1 and 2, are emitted from Windows 1 and 2, respectively.

Figure 1 illustrates the customized full-color light generation system, which consists of two 25 W D65 LED light sources (SAWS1566A, Seoul Semiconductor, Seoul, Republic of Korea) with a one-inch diameter [20]. This light source has a correlated color temperature of approximately 6500 K and a color rendering index greater than 90%. The emitted light passes through an 8 mm slit onto a 2.54 cm square concave reflection grating with a radius of 11.2 cm. The visible spectrum is produced by the light reflected from the grating and directed onto an electrically controlled 5.9-inch liquid crystal display (LCD, BOE Technology Group Co., Beijing, China). The spectral light is then switched by two separate LCD regions, each measuring 6 cm × 10 cm. Homemade integrators are employed, featuring an entrance pupil measuring 12 cm × 10 cm with a length of 12 cm. These integrators include a central partition with two 1.5 × 1.5 cm reflectors that help mix the spectral light uniformly before emission through two windows. Each window measures 1 cm × 1 cm and is observed at a distance of 25.4 cm, corresponding to a 2° angular subtense. This angle represents the optimal range for color vision, where cone cells are most densely concentrated at the center of the fovea. Increasing the angular subtense is unlikely to provide significant advantages, particularly for red-green discrimination, although it appears to have no notable effect in individuals with dichromacy [9]. The full-color light generation system can produce light that closely matches the CIE 1931 color space through the windows. In this study, 16 mixed spectral lights were evaluated using an micro spectrometer (FLAME-S-XR1-ES, Ocean Optics, Orlando, FL, USA) to verify that they align with the required spectrum as in the classic D-15 test.

### 2.4. Procedure for Conducting the CFCA Test

**Figure 2:**
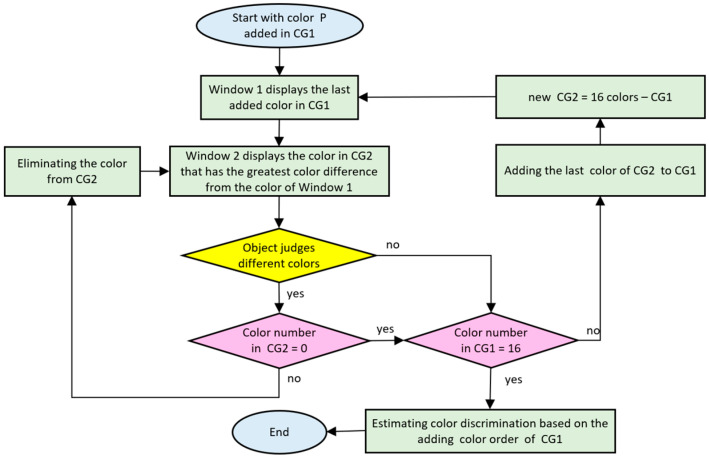
Flowchart of the customized CFCA procedure for evaluating CVD.

Figure 2 presents the flowchart of the proposed CFCA procedure. Initially, the pilot color P of the D-15 test is displayed in Window 1. The color shown in Window 1 constitutes color group 1 (CG1), while the remaining 15 colors form color group 2 (CG2). The color in CG2 with the greatest difference from the color in Window 1 is then displayed in Window 2. When the subject judges the two colors as different by pressing a key of the computer keyboard, that color is eliminated from CG2, and the next color with the maximum difference is displayed in Window 2. The comparison with Window 1 is complete when no colors remain in CG2 or when the subject judges the two colors as identical. At this point, the last color in CG2 is added to CG1 and displayed in Window 1. The remaining colors, excluding those in CG1, form the new CG2, and the process is repeated. This iterative comparison continues, with colors in both windows being replaced, until no new color can be added to CG1, i.e., when CG1 = 16 and CG2 = 0. Finally, the CFCA process estimates color discrimination according to the order in which colors are added to CG1. The final CFCA result is then compared with the classical D-15 method.

### 2.5. Data Analysis

**Figure 3:**
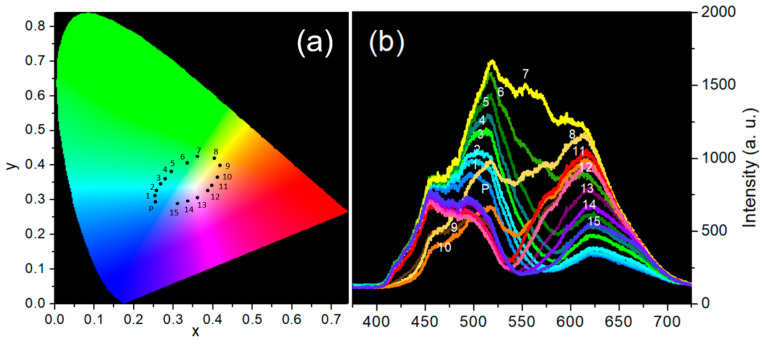
(**a**) CIE chromaticity diagram showing the 16 color discs labeled by P and 1–15; (**b**) Reflective spectra of the 16 color discs labeled by P and 1–15 irradiated by a D65 light.

This full-color light generation system ensures that the generated colors are identical to those reflected from the discs irradiated by the D65 light source. Figure 3a shows the 16 colors in the CIE 1931 color coordinate system, while Figure 3b presents the reflectance spectra of all 16 discs. In a CIE chromatic diagram, 16 points representing 16 discs are connected by black lines. The red, green, and blue lines represent the confusion lines (or confusion loci) for protans, deutans, and tritans, respectively. The letters P, D, and N indicate protan, deutan, and normal vision, respectively. Normal vision is characterized by few crossing lines, and the more severe the CVD, the greater the number of crossing lines. For quantitative data analysis, the chromaticities of the 16 discs are represented in the CIELUV color space [21]. Using the moment of inertia method, relative color difference vectors *V_n_* (*u_n_*, *v_n_*), where *n* = 1–15, illustrate the color differences between two disc coordinates connected by 15 black lines in the CIELUV color space [22,23]. The disc numbers associated with the head of each vector are indicated. If a rotation axis passes through the origin of the coordinate system, and the angle between this axis and the horizontal axis is *A*, then the distance from *V_n_* (*u_n_*, *v_n_*) to the rotation axis is given by

(1) $$y_{n}=v_{n}cosA-u_{n}sinA.$$

Assuming the *V_n_* (*u_n_*, *v_n_*) coordinate has a unit mass, the inertia is expressed as

(2) $$I=\sum {y_{n}}^{2},$$

which can be expanded as

(3) $$I={cos}^{2}A\sum {v_{n}}^{2}+{sin}^{2}A\sum {u_{n}}^{2}-2cosAsinA\sum u_{n}v_{n}.$$

Differentiating Equation (3) with respect to the angle *A* and setting the derivative to zero yields

(4) $$tan2A=\frac{\sum 2u_{n}v_{n}}{\sum \left({u_{n}}^{2}-{v_{n}}^{2}\right)}.$$

The two solutions for *A* lie in the range of −90° to 90°. Substituting these solutions into Equation (3) gives the maximum moment of inertia (*I_max_*) and the minimum moment of inertia (*I_min_*). The major radius (*R_maj_*) and minor radius (*R_min_*) are then obtained as follows:

(5) $$R_{\left(maj,min\right)}^{2}=\frac{\sum {y_{n}}^{2}}{n}=\frac{I_{\left(max,min\right)}}{n}.$$

The axis angle corresponding to *I_min_* is defined as the confusion angle (CA), along which the major radius (*R_maj_*) is plotted. The severity of the color defect is quantified by the confusion index (CI), defined as

(6) $$CI=\frac{R_{maj}\;of\;subject}{R_{maj}\;of\;error-free\;arrangement}$$

Thus, CVD can be quantitatively evaluated using the CA and CI values, which represent the type and severity of CVD, respectively. A CA threshold of 0.7° is applied to differentiate CVD types: angles greater than 0.7° indicate protan, angles below 0.7° indicate deutan, and angles less than −65° correspond to tritan. A CI of 1 to 1.6 is considered within the normal range, whereas a CI exceeding 1.6 indicates the presence of CVD traits.

The Pearson correlation analysis and Wilcoxon signed-rank test were performed using Statistical Package for the Social Sciences (SPSS, version 29) to examine differences and correlations between the CA and CI results obtained from the D-15 and CFCA methods. The Pearson product-moment correlation coefficient (r) is a statistical measure used to quantify the strength of a linear relationship between two variables [24]. The *r* value ranges from −1 to 1, where 0 indicates no correlation, and −1 or 1 indicates a perfect negative or positive correlation, respectively. In practice, an *r* value close to zero suggests a weak linear relationship, whereas larger absolute values of *r* correspond to stronger correlations. Linear relationships are commonly classified into three categories: low correlation (|*r*| < 0.4), moderate correlation (0.4 ≤ |*r*| < 0.7), and high correlation (0.7 ≤ |*r*|). The *p*-value in the Wilcoxon signed-rank test represents the probability of observing the data under the null hypothesis, which usually states that the median difference between paired observations is zero (i.e., there is no systematic difference between the two related groups) [25]. If the *p*-value is small (typically < 0.05), it indicates that the median difference is significantly different from zero, so one can reject the null hypothesis. If the *p*-value is large, there is no significant evidence to suggest a difference from zero.

## 3. Results

### 3.1. Full-Color Light Generation System in the CFCA Method

The conventional D-15 test requires arranging colored discs from P and 1–15 in the CIE 1931 color coordinate system (see Figure 3a). The 16 reflective spectra of the P and 1–15 discs irradiated by D65 light (see Figure 3b) can be reconstructed and projected through Windows 1 and 2 using the developed full-color light generation system (see Figure 1). The colors emitted from the two windows can be immediately changed by the customized software when the participant presses a specific key to indicate that the two colors differ, or waits 3 s to indicate that they are the same. The test is easy to operate for both children and the elderly. However, since the light is attenuated by the grating, LCD, and integrator, the color brightness appears somewhat low, approximately 50 lux in illuminance. All subjects completed the examinations within 5 min using our system, and none reported discomfort or difficulties during the CFCA test.

### 3.2. Normal Trichromats Assessed with the D-15 and CFCA Methods

**Figure 4:**
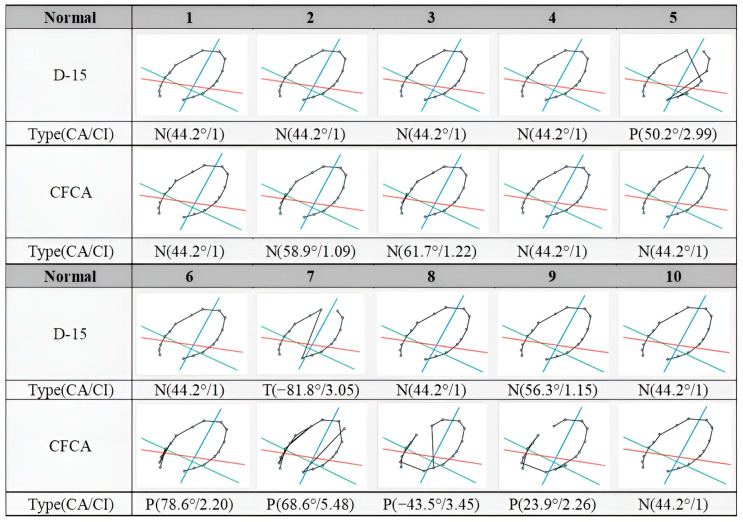
Comparisons of D-15 and CFCA test results from 10 normal trichromats (evaluated based on the Ishihara test). Each chromatic diagram contains 16 color points connected by black lines. The red, green, and blue lines indicate the confusion lines for protans, deutans, and tritans, respectively. The letters P, D, and N denote protan, deutan, and normal vision types.

For normal trichromats, the standard values of CA and CI are 44.2° and 1, respectively, with no cross lines observed among discs P and 1–15 in the corresponding chromatic diagram. Figure 4 presents the results of normal trichromats assessed with the D-15 and CFCA methods. In the D-15 dataset, there were eight cases of normal color vision, one case of protan, and one of tritan. Subjects 5 and 7 passed the Ishihara test but exhibited elevated CI values exceeding 1.6 in the D-15 test. The average CI, excluding Subject 7, was 1.24 with a standard deviation (SD) of 0.66, whereas Subject 7 had markedly higher CI values of 3.5 and 5.48 in the D-15 and CFCA tests, respectively, indicating severe CVD in the protan type. In the CFCA test, the dataset included six normal cases, three protans, and one deutan. Although Subjects 2 and 3 showed cross lines, their CI values (1.09 and 1.22) remained within the range typical for normal trichromats. Subjects 6–9 exhibited CI values greater than 1.6. Some participants classified as normal in the D-15 test demonstrated CVD-like characteristics in the CFCA test. The average CI was 1.58 with a SD of 0.87 in the CFCA test, higher than the average CI of 1.24 observed in the D-15 test.

### 3.3. Patients with CVDs Assessed with the D-15 and CFCA Methods

**Figure 5:**
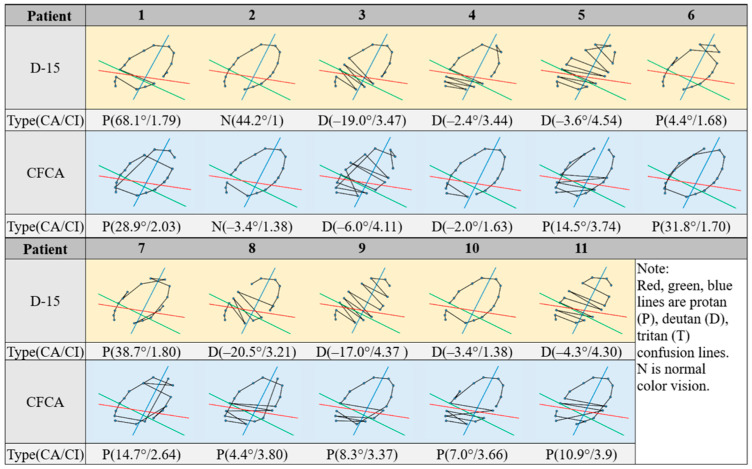
Comparisons of D-15 and CFCA test results from 11 subjects with CVDs (evaluated based on the Ishihara test). Each chromatic diagram contains 16 color points connected by black lines. The red, green, and blue lines indicate the confusion lines for protans, deutans, and tritans, respectively. The letters P, D, and N denote protan, deutan, and normal vision types.

Figure 5 shows the results of the D-15 and CFCA tests in 11 patients with CVDs. Except for Subject 2 in the D-15 test, all patients exhibited crossed lines, confirming the presence of CVD. Notably, Subject 2 failed the Ishihara test but passed the D-15 test. This outcome is consistent with the fact that the D-15 test passes 35–50% of deuteranomalous trichromats and over 40% of protanomalous trichromats [26,27]. This subject was identified as the deutan type in the CFCA test. In the D-15 test, the CI values ranged from 1 to 4.54, comprising three protans, seven deutans, and one normal subject. In the CFCA test, the CI values ranged from 1.38 to 4.11, with the dataset consisting of eight protans and three deutans. These values are listed in Table 2, together with the calculated mean ± SD and interquartile range (IQR) values. As indicated, the mean CI values for the D-15 and CFCA methods are 2.81 and 2.91, respectively. When two patients (Patients 4 and 10), who exhibited clear experimental deviations, were excluded due to large discrepancies between CI values in the D-15 (3.44 and 1.38) and CFCA (1.63 and 3.66) tests, the mean CI values for the two methods are closer, being 2.91 and 2.96, respectively. These discrepancies are more than 20 times greater than the difference between the mean CI values of the two methods (approximately 0.1, as shown in Table 2) and could serve as a criterion for statistical exclusion.

### 3.4. Statistical Analysis Between Two Tests

**Figure 6:**
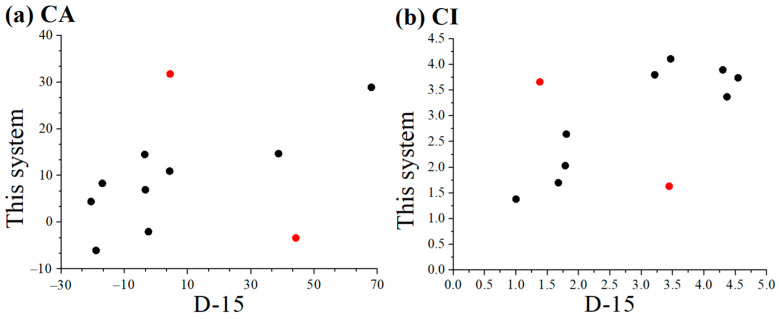
Pearson correlation analyses in (**a**) CA and (**b**) CI values between the D-15 test and the present CFCA method. Red dots represent data to be excluded in calculating *r*..

Pearson correlation analyses and Wilcoxon signed-rank tests were performed to evaluate differences and correlations in CA and CI values between the D-15 test and the CFCA method in the 11 patients. As shown in Figure 6, the Pearson correlation coefficients (*r*) were 0.41 for CA and 0.595 for CI. As described above, when two patients (Patients 4 and 10, as shown in red dots in the figure) were excluded, the *r* values increased to 0.821 for CA and 0.884 for CI. This suggests that these two methods are highly correlated.

**Figure 7:**
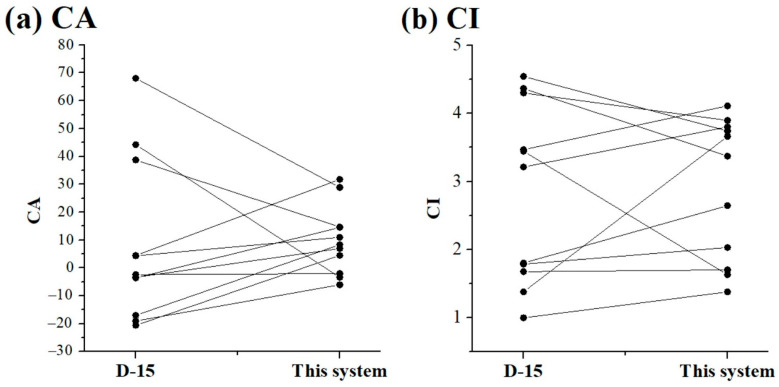
Wilcoxon signed-rank tests in (**a**) CA and (**b**) CI values between the D-15 test and the present CFCA method.

Figure 7 illustrates the results of the Wilcoxon signed-rank tests. In general, *p*-values below 0.05 indicate a significant difference, whereas values above 0.05 are considered not significant. In this case, the calculated *p*-values for CA and CI (0.688 and 0.587, respectively) indicate no significant differences. Therefore, it can be concluded that the conventional D-15 test and the CFCA system exhibit no substantial discrepancy, and their results can be used interchangeably. The present system offers several advantages, including ease of use, expandable color options (beyond the 16 discs in the D-15 test), and, most importantly, the ability to acquire a personalized visual spectrum for designing customized colorblind glasses.

## 4. Discussion

### 4.1. The CFCA Method with Full-Color Light Generation System

Although the process of “judging different colors” in Figure 2 appears somewhat intricate, the task itself is straightforward: the subject simply presses a response key within 3 s to indicate whether two colors are different. When the colors are the same, the participant waits 3 s until the program automatically advances to a different color stage. This 3 s duration can be adjusted according to the participant’s physical condition (e.g., elderly individuals or children). Although the judging time accumulates as the number of colors in CG1 increases from 1 to 16 across the 16 color discs, the total test duration remains under 5 min. Moreover, a stage is considered complete once the subject judges the two colors as the same, even if CG2 > 0 at that stage. Therefore, the color set in each subsequent stage is determined by the comparison responses obtained in the previous stage.

### 4.2. Assessment of Normal Trichromats

In evaluating normal trichromats, the average CI for the D-15 test is 1.24, whereas the CFCA test yields an average CI of 1.58. These statistical results highlight the reliability of the CFCA method and demonstrate its sensitivity and accuracy in detecting color vision abnormalities.

### 4.3. Assessment of CVD Patients

For the 11 CVD patients, the Pearson correlation test indicated a significant correlation for both CA and CI between the examinations conducted using the D-15 test and the CFCA system. The corresponding *p*-values between the two tests were 0.688 for CA and 0.587 for CI. In general, a *p*-value below 0.05 indicates a significant change, whereas a value above 0.05 indicates no significant change. Since both of the *p*-values are above 0.05, no significant differences were observed. Consequently, it can be concluded that the CFCA and D-15 produce interchangeable results. However, the non-significant result may also be due to the low statistical power resulting from the small sample size. In addition, it is worth noting that, as shown in Figure 4 and Figure 5, clear discrepancies exist between the D-15 and CFCA results, suggesting the possibility of group contamination (e.g., CVD patients included in the normal group and vice versa). Therefore, participants should be evaluated using the gold standard method, the Nagel Anomaloscope.

### 4.4. Comparison with Other Methods for Identifying CVDs

Table 3 lists a comparison of general methods for identifying CVDs [28,29,30]. The rapid development of computerized color-vision assessments is driven by progress in computer and monitor technologies. For example, Waggoner’s computerized color-vision (CCV) test has a computer version of D-15 (WC-D15) [31,32], and the Ishihara test. These tests can be installed on computers or smartphones. The WC-D15 examination method is similar to the D-15 test, except that all disc movements are changed to image-dragging movements on the screen. Almustanyir et al. reported identical results for D-15 and WC-D15 tests [31,32]. However, the screen quality can also affect the colors presented. The CCV test employs a light-emitting diode (LED) screen to display metameric colors; the screen generally uses three different color LEDs and cannot comprise a full-color gamut.

In this study, two LCD light sources are employed in the customized system. The high-ranking LED light corresponds to a true chroma within a full-color gamut generated by a non-metameric light source. The FM-100 or D-15 test requires illumination at 6500 K with high color rendering from the environment. The full-color light generation system developed in this study is capable of producing various colors. Compared with the classic D-15 test, where the reflected light from the discs is influenced by the direction and distance of illumination, the CFCA system allows subjects to directly view light at an illuminance of about 50 lux, thereby reducing the effects of the surrounding environment. Furthermore, as shown in Figure 1, the CFCA incorporates a unique algorithm for color display and arrangement. Different color sets are displayed in Windows 1 and 2 based on the subject’s prior responses to color sets. In other words, the proposed system prevents memorization effects. It should be noted that the differentiation time for each color set must be less than 3 s, which is shorter than the time control used in traditional methods. This limitation is necessary because individuals with CVDs may recognize colors if the control time exceeds 3 s, potentially leading to incorrect examination results. In the CFCA method, all 16 colors are ultimately arranged according to the subject’s two-color discrimination ability within a short testing period of 5 min. This duration is considerably shorter than the time and effort required for conventional FM-100 or D-15 tests.

In addition, the proposed system can provide quantitative results, such as those from the D-15 test, owing to its digitization and computational capabilities. Beyond the number of crossing lines indicated in CIE coordinates, the CA and CI parameters provide more accurate metrics for evaluating the type and severity of CVD [23]. In particular, CA can categorize each CVD type over a relatively wide angular range. CI, related to the number of crossing lines, serves as the primary diagnostic parameter for CVD because it identifies the type of color blindness when CA values are large. Although the Nagel anomaloscope test is considered the gold standard for CVD examination, it is not widely adopted due to its high cost and the limited range of color perception it can evaluate (primarily red–green, or blue–yellow, deficiencies). And unlike the complicated process and expense of the Nagel anomaloscope test, the proposed system and method are easy to learn and administer for both examiners and subjects. Furthermore, the CFCA system is simple to use, as the subject only needs to press a response key when judging that two different colors are displayed, and no action is required when the colors are the same. To prevent false negatives caused by random guessing (e.g., subjects pressing the key for all displays), identical color sets are randomly presented in Windows 1 and 2. This ensures that repeated incorrect responses for identical colors are detected. Compared with the fixed number of colors presented in the Ishihara test, the colors in the proposed system are displayed in a sequence determined by our software, thereby minimizing learning effects.

The full-color light generation system incorporates a transparent LCD to program and control spectral transmission. The LCD transmits only about 10% in the visible region, with particularly low transmittance at short wavelengths. The 25 W D65 LED light source has reduced intensity in the purple and red regions, which can be enhanced by supplementary LEDs. Although the CFCA method was applied in this study, the full-color light generation system could not fully match the spectra in Figure 3b because of the weak intensity in certain regions. This may explain the larger SD values observed in Section 3.2. To address this, we plan to modify the system using multiple LEDs with central wavelengths distributed across the visible spectrum. Increasing the spectral intensity is expected to improve the accuracy of CVD examination results. Moreover, the sample size is relatively small, which limits the generalizability of the system. Despite some limitations, personalized notch filters as colorblind glasses can be designed by acquiring a personalized visual spectrum via this system. Relevant data are being prepared for publication in a separate study.

## 5. Conclusions

The developed full-color light generation system with CFCA provides desired color-vision assessments, with results that are highly consistent with those of the conventional D-15 test. Therefore, the proposed system can be applied conveniently and efficiently in patients with CVDs. In addition, the system can rapidly perform the anomaloscope test, the FM-100, or any two-color discrimination study. We are continuously improving our prototype to achieve higher spectral intensity and finer control, which will further enhance the accuracy of CVD examinations. Furthermore, we have designed notch filters tailored for patients with CVDs using this system.

## Figures and Tables

**Table 1:** Demographic and health status information of all participants. (F: female; M: male; HTN: hypertension; DM: diabetes mellitus).

Normal	Sex	Age	HTN *	DM *	Patient	Sex	Age	HTN *	DM *
1	F	27	No	No	1	M	39	No	No
2	F	59	No	No	2	M	19	No	No
3	F	29	No	No	3	M	29	No	No
4	F	26	No	No	4	M	26	No	No
5	F	29	No	No	5	M	16	No	No
6	F	41	No	No	6	M	19	No	No
7	F	29	No	No	7	M	9	No	No
8	F	25	No	No	8	M	25	No	No
9	F	26	No	No	9	M	24	No	No
10	F	28	No	No	10	M	25	No	No
					11	M	68	No	No

* reported by the participant during consultation with an ophthalmologist.

**Table 2:** Confusion angle (CA) and confusion index (CI) values for 11 patients evaluated with both D-15 and computerized full-color assessment (CFCA) methods.

Patient	D-15			CFCA		
	CA	CI	Type	CA	CI	Type
1	68.05°	1.79	Protan	28.88°	2.03	Protan
2	44.22°	1	Normal	−3.36°	1.38	Normal
3	−18.98°	3.47	Deutan	−6.04°	4.11	Deutan
4	−2.38°	3.44	Deutan	−2.03°	1.63	Deutan
5	−3.58°	4.54	Deutan	14.51°	3.74	Protan
6	4.44°	1.68	Protan	31.75°	1.70	Protan
7	38.73°	1.80	Protan	14.69°	2.64	Protan
8	−20.55°	3.21	Deutan	4.45°	3.80	Protan
9	−16.96°	4.37	Deutan	8.32°	3.37	Protan
10	−3.36°	1.38	Normal	6.95°	3.66	Protan
11	−4.27°	4.30	Deutan	10.95°	3.90	Protan
Mean with SD	7.76 ± 29.3	2.81 ± 1.32		9.92 ± 12.2	2.91 ± 1.01	
IQR	55.69	2.62		16.72	2.1	
Mean with SD excluding 4 and 10	10.1 ± 32.2	2.91 ± 1.36		11.6 ± 12.8	2.96 ± 1.05	
IQR excluding 4 and 10	59.45	2.6		21.24	2.0	

**Table 3:** Comparison of general methods for identifying color vision deficiencies.

Detection Method Comparison	Nagel Anomaloscope †	Ishihara	FM-100	D-15	CCV	CFCA
Published year	1907	1917	1940s	1940s	2010s	2023
Full-color gamut	No	Possible ^$^	Possible ^$^	Possible ^$^	Not Fully	Yes
Non-metameric light	Unnecessary	Possible ^$^	Possible ^$^	Possible ^$^	No	Yes
Time control	No	No	No	No	Possible	Yes
Short test time	No	Yes	No	Possible	Yes	Yes
Digitization	Possible	Poor	Poor	Poor	Yes	Yes
Non-memorable	Yes	Poor	Yes	Yes	Yes	Yes
Easy to use	Poor	Possible *	Poor	Poor	Possible	Yes
False negative prevention	Possible	Poor	Poor	Poor	Possible	Yes

† Gold standard; ^$^ Required D65 light irradiation; * Required professional interpretation if the tester is color blind.

## Data Availability

The original contributions presented in this study are included in the article. Further inquiries can be directed to the corresponding author.
